# Mental burden and moral distress among oncologists and oncology nurses in Germany during the third wave of the COVID-19 pandemic: a cross-sectional survey

**DOI:** 10.1007/s00432-023-04580-x

**Published:** 2023-01-26

**Authors:** Sabine Sommerlatte, Celine Lugnier, Olaf Schoffer, Patrick Jahn, Anna-Lena Kraeft, Eleni Kourti, Patrick Michl, Anke Reinacher-Schick, Jochen Schmitt, Thomas Birkner, Jan Schildmann, Stephan Herpertz

**Affiliations:** 1 Faculty of Medicine, Institute for History and Ethics of Medicine, Interdisciplinary Centre for Health Sciences, Martin Luther University Halle-Wittenberg, Halle (Saale), Germany; 2 Department of Hematology, Oncology and Palliative Care, St. Josef Hospital, Ruhr University Bochum, Bochum, Germany; 3 Center for Evidence-Based Healthcare, University Hospital Carl Gustav Carus and Carl Gustav Carus Faculty of Medicine, Technische Universität Dresden, Dresden, Germany; 4 Health Services Research Working Group, Department of Internal Medicine, University Hospital Halle (Saale), Halle (Saale), Germany; 5 Department of Medicine, Internal Medicine IV, University Hospital Heidelberg, Heidelberg, Germany; 6 Department of Psychosomatic Medicine and Psychotherapy, LWL-University Clinic, Ruhr University Bochum, Bochum, Germany

**Keywords:** COVID-19, Oncology, Mental burden, Moral distress, Nurses, Physicians

## Abstract

**Purpose:**

There is evidence for mental burden and moral distress among healthcare workers during the pandemic. However, there is scarcity of analyses regarding possible correlations of mental burden and moral distress in this context. This study provides data to quantify mental burden and possible associations with moral distress among physicians and nurses working in oncology in Germany.

**Methods:**

We conducted a cross-sectional online survey with physicians and nurses working in oncology in Germany between March and July 2021. Next to sociodemographic characteristics and working conditions, mental burden and moral distress were assessed using standardized instruments. Binary multivariate logistic regression using the enter method was performed in order to explore the relationship between mental burden and moral distress.

**Results:**

121 physicians and 125 nurses were included in the study. Prevalence of clinically relevant depressive symptoms, anxiety, somatic symptoms, burnout symptoms and moral distress was 19.2, 14.5, 12.7, 46.0 and 34.7% in physicians and 41.4, 24.0, 46.8, 46.6 and 60.0% in nurses respectively. Mental burden was significantly associated with moral distress, being female/diverse, younger age < 40 and increase in workload. Nurses who felt sufficiently protected from COVID-19 reported significantly less moral distress.

**Conclusion:**

To improve pandemic resilience, there is a need to ensure safe working environment including psychosocial support. Further evidence on risk and protective factors for moral distress is needed to be able to develop and implement strategies to protect healthcare workers within and beyond the pandemic.

**Supplementary Information:**

The online version contains supplementary material available at 10.1007/s00432-023-04580-x.

## Introduction

In the wake of the COVID-19 pandemic, the social importance of the healthcare workforce, on the one hand, and the increasing shortage of staff in the healthcare sector, on the other hand, have once again come into sharp focus (Schmedt 2020). There is evidence for high mental burden on healthcare workers within the context of the outbreak of SARS-CoV-2 and staff turnover associated with mental burden during the pandemic (Morawa et al. 2021; Schug et al. 2022; Stefanovska—Petkovska et al. 2021; Tabur et al. 2022). Healthcare professionals in oncology caring for high-risk patients with life-threatening diseases have been affected in particular by additional challenges during the pandemic. A large longitudinal study conducted by the European Society for Medical Oncology (ESMO) Resilience Task Force with more than 1000 oncology professionals from over 100 countries points to a major impact of the pandemic on mental health, wellbeing and job performance in oncology, displaying an increase in burnout and distress during the pandemic (Banerjee et al. 2021; Lim et al. 2022). The study further revealed that in February/March 2021, 38% (*n* = 100/266) thought about leaving the profession (Lim et al. 2022). A number of risk factors such as being female, younger age (≤ 40 years) and changes in working hours have been identified to be associated with higher mental burden in oncology (Banerjee et al. 2021). Furthermore, belonging to the nursing profession has been shown to be a risk factor for mental burden in healthcare workers in the context of the pandemic (Kramer et al. 2021). Next to studies on mental health, burnout and job-abandonment, there has been an increasing interest in moral distress of healthcare workers (Riedel et al. 2022; Sheather and Fidler 2021). Moral distress arises “when one knows the right thing to do, but institutional constraints make it nearly impossible to pursue the right course of action” (Jameton 1984). Inadequate staffing and increased staff turnover as well as inadequate understanding regarding the situation have been shown to contribute to moral distress (Hamric et al. 2012). Against this background, it seems likely that pandemic-related limitations in healthcare such as shortages of protective clothing, treatment postponements, illness, or quarantine, might further increase the risk for moral distress in oncology healthcare workers (Hlubocky et al. 2021). Fittingly with this assumption, a recent German study with 3293 healthcare workers from different specialties, which was conducted around the so-called “first wave” in 2020, showed an increase in moral distress compared to reference samples before the pandemic (Schneider et al. 2021). However, there is scarcity of data regarding the extent to which moral distress during the pandemic contributes to mental burden among oncology professionals. This is particularly true for the situation in Germany given the scarcity of comprehensive data to quantify mental burden and moral distress on physicians and nurses working in oncology. Therefore, the aim of this study was to assess mental burden and moral distress among oncology physicians and nurses in Germany by means of standardized instruments. Our analyses focus on the relationship between mental burden and moral distress as well as sociodemographic factors and working conditions associated with particular high mental burden and moral distress.

## Materials and methods

### Study design and participants

We conducted a cross-sectional online survey with oncology physicians and nurses in Germany between March and July 2021 during the so-called “third wave” of the COVID-19 pandemic. Data collection was done via the platform Lime-Survey, which is located on the server of Martin Luther University Halle-Wittenberg. Physicians and nurses were recruited via mailing lists among members of Working Group for Medical Oncology of the German Cancer Society (Arbeitsgemeinschaft Internistische Onkologie, AIO, *n* = 929) and members of Oncology Nursing and Pediatric Nursing Conference (Konferenz Onkologischer Kranken- und Kinderkrankenpflege, KOK, *n* = 1750) of the German Cancer Society respectively. E-mails with a survey link as well as a description of the study and its aims were sent to all physicians (March 22, 2021) and nurses (May 4, 2021) in the respective mailing list, followed by a reminder. Both surveys were closed on July 7, 2021. Study participation was voluntary and anonymous. Participants were asked to provide a code consisting of the first two letters of their mother's first name, their mother's month of birth, and the number of siblings, which allowed to identify possible duplicates.

### Measures

#### Sociodemographic data and working conditions

Participants completed a sociodemographic online questionnaire including the following items: age, gender, years of work experience and occupational setting (inpatient/outpatient/other setting). Nurses completed additional questions on working conditions, which were either rated on a 5 point scale from 0 (I strongly disagree) to 4 (I strongly agree) or with yes/no/I do not know.

#### Moral distress

Moral distress was assessed using a German version of the Moral Distress Thermometer (MDT). The MDT has been adapted by Mehlis et al. according to the validated German Version of the Cancer Distress Thermometer (CDT) of the National Comprehensive Cancer Network (NCCN), which the development of the MDT was initially based on. Participants were provided with a definition of moral distress (“Moral distress occurs when you think you know what is right to do. However, a circumstance or a person prevents you from doing the right thing.”) and asked to rate the level of experienced moral distress on a 11 point Likert scale ranging from 0 (no moral distress) to 10 (worst possible distress) (Mehlis et al. 2018; Wocial and Weaver 2013). Analogous to the CDT, a cutoff score of  ≥ 5 was applied to identify participants having high levels of moral distress (Mehnert et al. 2006).

Mental burden was operationalized by measuring depressive symptoms, anxiety and somatization/somatic symptom severity as well as burnout symptoms and pandemic stress load.

#### Depressive symptoms

Depressive symptoms were assessed using the German version of the Patient Health Questionnaire-9 (PHQ-9). It consists of 9 questions on the frequency of depressive symptoms, which have to be answered on a 4 point Likert scale ranging from 0 (not at all) to 3 (almost every day). A sum score (0–27) was calculated. Cutoff points of  ≥ 5, ≥    10, ≥ 15   and   ≥ 20 were applied to identify mild, moderate, moderately severe and severe depressive symptoms, respectively (Kroenke et al. 2001). A cutoff ≥ 10 was applied to identify clinically relevant depressive symptoms (Kroenke and Spitzer 2002). The questionnaire has good validity and internal consistency (Cronbach's *α* = 0.88) (Gräfe et al. 2004). In terms of criterion validity, the PHQ-9 has proven to be superior compared to other questionnaires (Löwe et al. 2004).

#### Anxiety

For assessment of anxiety, the German version of the Generalized Anxiety Disorder Scale (GAD-7) was used. It contains 7 items, assessing the frequency of symptoms of general anxiety disorder on a 4 point Likert scale ranging from 0 (not at all) to 3 (almost every day). A sum score (0–21) was calculated. Cutoff scores of  ≥ 5,   ≥ 10 and ≥ 15 were applied to identify symptoms of mild, moderate and severe anxiety respectively (Löwe et al. 2008). A cutoff  ≥ 10 has a sensitivity of 89% and a specificity of 82% for the diagnosis of GAD and was applied to detect clinically relevant anxiety. The questionnaire is valid and shows good reliability (Löwe et al. 2008; Spitzer et al. 2006).

#### Somatic symptom severity

The German version of the PHQ-15 was used to measure somatic symptom severity. It comprises questions on 15 somatic symptoms, each of which is rated from 0 (not bothered at all) to 2 (bothered a lot). Cutoff scores of  ≥ 5, ≥  10 and ≥  15 represent low, medium and high somatic symptom severity. A cutoff ≥   10 was applied to identify clinically relevant somatic symptoms. The questionnaire shows good validity and reliability (Cronbach’ s *α* = 0.80) (Kroenke et al. 2002).

#### Burnout

The German Version of the Maslach Burnout Inventory (MBI) was applied to assess symptoms of burnout (Maslach and Jackson 1981). The questionnaire consists of 22 items, assessing three dimensions of burnout (emotional exhaustion (EE), depersonalization (DP) and personal accomplishment (PA)) on a 7-point Likert scale ranging from 0 (never) to 6 (every day). Existence of burnout symptoms was assumed when a participant displayed high levels of EE (≥ 27), high levels of DP (≥ 10) or low levels of PA (< 33) (Grunfeld et al. 2000; Shanafelt et al. 2012).

#### Pandemic stress load

Pandemic stress load was assessed via “FACT-19 questionnaire for the assessment of pandemic COVID-19-stress levels” (Bering R, Eckhard A, Schedlich C, & Zurek, G (2020) FACT-19 Fragebogen zur Erfassung der pandemischen COVID-19 Stressbelastung (unpublished)). The FACT-19 questionnaire is a newly developed instrument that is in part based on the International Classification of Functioning, Disability and Health (ICF) and operationalizes pandemic stress levels in a triangular model containing 1. pre-pandemic risk factors, 2. (sources of) acute pandemic stress and 3. context factors. A sum score was calculated for pre-pandemic risk factors and acute pandemic stress respectively, with higher scores indicating higher stress levels. In addition, acute pandemic stress is also broken down in terms of four different sources of occurrence, (A) lethal threat, (B) existential fear, (C) isolation and (D) fear dynamics (Eckhard et al. 2021).

### Statistics

Statistical analyses were conducted with IBM SPSS Statistics version 28.0 for Windows. Descriptive statistics (means and standard deviations, medians with interquartile range, absolute and relative frequencies and Clopper–Pearson confidence intervals) were calculated to describe sociodemographic characteristics, working conditions and levels of mental burden. Percentages are reported in relation to the number of valid responses.

Since we assume non-linearity in our data, binary multivariate logistic regression using the enter method was performed for nurses and physicians separately in order to explore the relationship between mental burden and moral distress and to determine epidemiological factors and working conditions associated with higher levels of mental burden and moral distress. To investigate the association of depressive symptoms and moral distress with professional group (i.e., nurses or physicians), additional models were calculated for nurses and physicians jointly. To assess the robustness of the results, linear regression analysis was conducted additionally for metric variables. For logistic regression analysis, odds ratios (OR) and their 95% confidence intervals (CI) were calculated. Depressive symptoms, anxiety, somatic symptom severity, burnout symptoms, and moral distress were set as dependent variables. Moral distress was dichotomized using a cutoff ≥  5 (Mehnert et al. 2006). For depressive symptoms, anxiety and somatic symptoms, a cutoff ≥ 10 was applied (Kroenke and Spitzer 2002; Kroenke et al. 2002; Spitzer et al. 2006). Gender, age, occupational field, profession and moral distress were set as independent variables. In the models calculated separately for nurses, the answers to the questions on increase in workload, staff shortages, difficulties in building relationships with patients, access to protective clothing, subjective feeling of sufficient protection, vaccination status and number of confirmed COVID-19 cases in employees and patients in the last 4 weeks were additionally included as independent variables. In order not to obtain too many independent variables, answers to ordinally scaled questions were dichotomized by putting “strongly disagree”, “rather disagree” and “partly agree partly disagree” under one category and “rather agree” and “strongly agree” under a second category. Since according to the literature, age < 40 is a risk factor for mental burden on health care workers, the variable was dichotomized using a cutoff ≥ 40 years (Kramer et al. 2021). Since only one person identified as diverse, the attributes “female” and “diverse” were combined under one category. Due to low response rates, results of FACT-19 were not included in the models. A level of significance of *p* < 0.05 was predetermined for all analyses.

## Results

### Sample characteristics

Of the 929 physicians and 1750 nurses in the mailing lists, 148 physicians and 170 nurses accessed the surveys. Blank responses (physicians *n* = 24, nurses *n* = 42), duplicates (same code, age, and gender; physicians *n* = 2, nurses *n* = 3) and answers of persons not belonging to the medical or nursing profession (physicians *n* = 1) as well as single incomplete questionnaires (Table 2) were removed from the data set. Ultimately, responses of 121 physicians and 125 nurses were included in the study. Overall response rate was 13 and 7% for physicians and nurses, respectively. Sample characteristics are shown in Table 1.

**Table 1:** Sample characteristics

	Physicians	Nurses
Gender		
Male *n* (%)	69 (57.0)	23 (18.4)
Female *n* (%)	51 (42.2)	102 (81.6)
Diverse *n* (%)	1 (0.8)	0 (0)
Age		
Mean ± SD	47.4 ± 10.6	43.7 ± 11.8
Years of work experience		
Mean ± SD	19.9 ± 10.9	22.3 ± 12.2
Occupational field		
Inpatient *n* (%)	69 (57.0)	85 (68.0)
Outpatient *n* (%)	49 (40.5)	32 (25.6)
Other *n* (%)	3 (2.5)	8 (6.4)

*SD* standard deviation

### Mental burden moral distress and working conditions

**Fig. 1:**
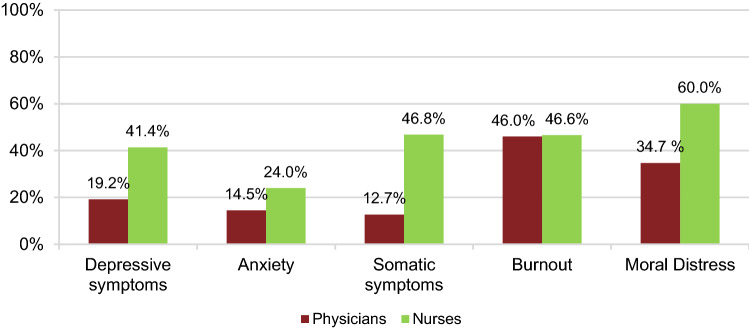
Prevalence of mental burden and moral distress in physicians and nurses. 19.2% (CI 13– 27%) of physicians and 41.4 (CI 33–51%) of nurses reported clinically relevant depressive symptoms (PHQ-9 ≥ 10). Clinically relevant anxiety was identified in 14.5% (CI 9– 22%) physicians and 24.0% (CI 17–33%) nurses (GAD-7 ≥ 10). Clinically relevant somatic symptoms were reported by 12.7% (CI 7–20%) physicians and 46.8% (CI 37–57%) nurses (PHQ-15 ≥ 10). 46.0% (CI 36–56%) of the physicians and 46.6% (37–57%) of the nurses reported symptoms of burnout in at least one subscale of the MBI. 42 physicians (34.7%; CI 26–44%) and 75 nurses (60.0%; CI 51–69%) reported moral distress ≥ 5

**Fig. 2:**
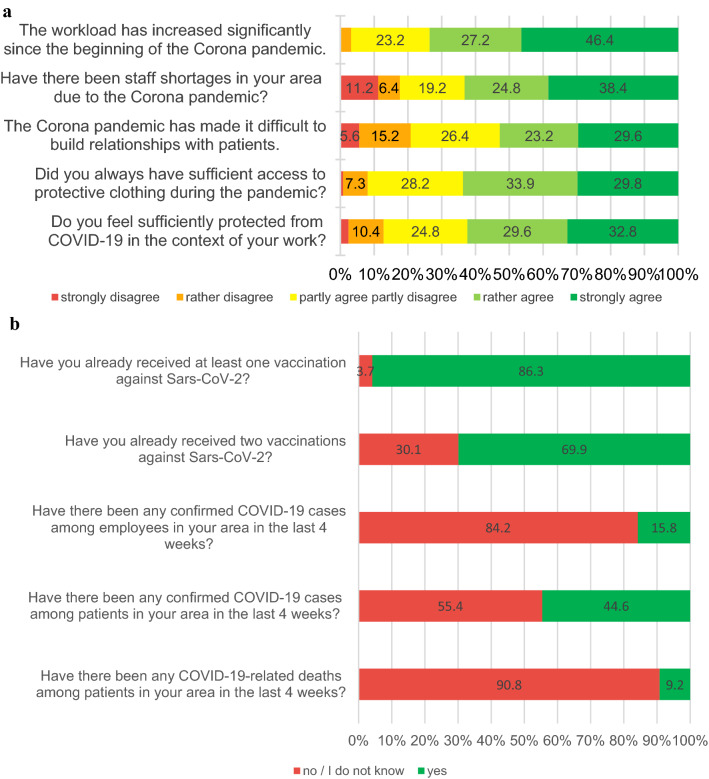
**a** Working conditions for nurses: workload, staff shortages, relationship building with patients, protection from COVID-19. **b** Working conditions for nurses: vaccination status, confirmed COVID-19 cases, COVID-19-related deaths

Descriptive statistics of mental burden and moral distress are shown in Table 2. Prevalences are shown in Fig. 1 and Table 3. Nurses displayed higher mental burden than physicians with regard to all assessed outcomes. With regard to acute pandemic stress load, the sources of origin C (isolation) and D (fear dynamics) dominate among both groups (Table 2). The survey among nurses included additional questions on working conditions (Fig. 2a, b). Increase in workload was attributed to shortage of staff (80%), hygiene requirements (84.8%) and loss of support by patients’ relatives (56.8%). Of the 17 persons, who had not received a vaccination yet, 11 planned to get vaccinated, 3 did not plan to get vaccinated and 3 did not know yet.

**Table 2:** Descriptive statistics of mental burden and moral distress

	*n*	Mean (± SD)	Median (IQR)	Range
Moral distress				
Physicians				
Completed	121	3.83 (± 2.36)	3.00	10
Missing	0	–	–	–
Nurses				
Completed	125	5.09 (± 2.26)	5.0	9
Missing	0	–	–	–
Depressive symptoms				
Physicians				
Completed	120	6.14 (± 5.21)	5.00 (5.00)	26
Missing	1	–	–	–
Nurses				
Completed	123	8.37 (± 5.00)	7.00 (7.00)	21
Missing	2	–	–	–
Anxiety				
Physicians				
Completed	117	5.14 (± 4.40)	4.00 (5.00)	20
Missing	4	–	–	–
Nurses				
Completed	121	6.55 (± 4.40)	5.00 (5.00)	18
Missing	4	–	–	–
Somatic symptoms				
Physicians				
Completed	110	4.80 (± 4.27)	4.00 (5.00)	18
Missing	11	–	–	–
Nurses				
Completed	111	9.79 (± 5.87)	9.00 (9.00)	26
Missing	14	–	–	–
Burnout symptoms–emotional exhaustion				
Physicians				
Completed	100	20.56 (± 11.92)	19.00 (17.75)	52
Missing	21	–	–	–
Nurses				
Completed	103	21.98 (± 12.17)	22.00 (19.00)	49
Missing	22	–	–	–
Burnout symptoms–depersonalization				
Physicians				
Completed	100	6.41 (± 4.86)	6.00 (6.75)	21
Missing	21	–	–	–
Nurses				
Completed	103	4.99 (± 4.53)	4.00 (6.00)	18
Missing	22	–	–	–
Burnout symptoms–personal accomplishment				
Physicians				
Completed	100	39.68 (± 5.64)	41.00 (6.00)	27
Missing	21	–	–	–
Nurses				
Completed	103	37.55 (± 5.76)	38.00 (7.00)	23
Missing	22	–	–	–
FACT-19: pre-pandemic risk factors				
Physicians				
Completed	88	1.91 (± 1.71)	1.00 (2.00)	7.0
Missing	33	–	–	–
Nurses				
Completed	92	2.55 (± 2.05)	2.00 (3.00)	9
Missing	33	–	–	–
FACT-19: acute pandemic stress				
Physicians				
Completed	88	6.06 (± 2.45)	5.50 (3.38)	13.5
Missing	33	–	–	–
Nurses				
Completed	92	7.14 (± 2.71)	6.75 (3.88)	12.5
Missing	33	–	–	–
FACT-19: acute pandemic stress source A (lethal threat)				
Physicians				
Completed	88	0.22 (± 0.65)	0.00 (0.00)	3.5
Missing	33	–	–	–
Nurses				
Completed	92	0.49 (± 1.15)	0.00 (0.00)	4.5
Missing	33	–	–	–
FACT-19: acute pandemic stress source B (existential fear)				
Physicians				
Completed	88	0.56 (± 0.66)	0.00 (1.00)	3.0
Missing	33	–	–	–
Nurses				
Completed	92	0.58 (± 0.73)	0.00 (1.00)	3.0
Missing	33	–	–	–
FACT-19: acute pandemic stress source C (isolation)				
Physicians				
Completed	88	1.64 (± 0.90)	2.00 (1.00)	4.0
Missing	33	–	–	–
Nurses				
Completed	92	1.83 (± 0.66)	2.00 (0.00)	3.0
Missing	33	–	–	–
FACT-19: acute pandemic stress source D (fear dynamics)				
Physicians				
Completed	88	2.55 (± 1.28)	2.50 (2.00)	5.5
Missing	33	–	–	–
Nurses				
Completed	92	3.30 (± 1.40)	3.25 (1.88)	6.5
Missing	33	–	–	–

*SD* standard deviation, *IQR* interquartile range

**Table 3:** Prevalence of mental health burden on physicians and nurses

	Prevalence	95% CI
Physicians		
Mild depressive symptoms	35.8%	27–45%
Moderate depressive sympotoms	11.7%	7–19%
Moderately severe depressive symptoms	4.2%	1–10%
Severe depressive symptoms	3.3%	1–8%
Mild anxiety	29.1%	21–38%
Moderate anxiety	10.3%	5–17%
Severe anxiety	4.3%	1–10%
Low somatic symptom severity	28.2%	20–38%
Medium somatic symptom severity	6.4%	3–13%
High somatic symptom severity	6.4%	2–13%
Symptoms of burnout	46.0%	36–56%
Nurses		
Mild depressive symptoms	35.0%	27–44%
Moderate depressive symptoms	27.6%	20–36%
Moderately severe depressive symptoms	11.4%	6–18%
Severe depressive symptoms	2.4%	1–7%
Mild anxiety	41.3%	32–51%
Moderate anxiety	17.4%	11–25%
Severe anxiety	6.6%	3–13%
Low somatic symptom severity	32.4%	24–42%
Medium somatic symptom severity	24.3%	17–33%
High somatic symptom severity	22.5%	15–31%
Symptoms of burnout	46.6%	37–57%

### Factors associated with mental burden and moral distress

According to the joint model of physicians and nurses, higher levels of depressive symptoms were significantly associated with being female/diverse and higher levels of moral distress. Moral distress was significantly associated with belonging to the nursing profession (Table 4). However, no significant association between professional group and depressive symptoms could be shown (Table 5). With regard to physicians alone, burnout symptoms were significantly associated with age < 40 (Table 6). Anxiety was significantly associated with moral distress (Table 7). Binary logistic regression for somatic symptom severity (Nagelkerke’s *R*^2^ = 0.13; *p* = 0.107; *n* = 110) did not yield a significant model. With regard to nurses alone, female participants and participants with higher scores of moral distress as well as greater increase in workload showed significantly higher levels of depressive symptoms (Table 8). Burnout symptoms were significantly associated with higher levels of moral distress (Table 9). Higher levels of moral distress in nurses were significantly associated with being female, whereas respondents who felt sufficiently protected from COVID-19 reported significantly less moral distress (Table 10). For anxiety (Nagelkerke’s *R*^2^ = 0.22; *p* = 0.173; *n* = 110) and somatic symptom severity (Nagelkerke’s *R*^2^ = 0.25; *p* = 0.064; *n* = 102), no significant models emerged. Results of linear regression analysis did not contradict those of logistic regression analysis (Supplementary tables 1 − 5).

**Table 4:** Logistic regression for moral distress in physicians and nurses

	B	SE	*p*	OR	95% CI for OR	
Age < 40	0.064	0.295	0.827	1.066	0.598	1.902
Being female/diverse	0.577	0.301	0.055	1.781	0.988	3.211
Occupational setting			0.930			
Hospital setting	–0.109	0.297	0.714	0.897	0.501	1.605
Other Setting	0.000	0.672	1.000	1.000	0.268	3.733
Professional group nursing	**0.834**	**0.291**	**0.004**	**2.303**	**1.301**	**4.076**
Constant	–0.847	0.302	0.005	0.429		

Statistically significant values are printed in bold

*B* regression coefficient, *SE* standard error, *OR* odds ratio

Nagelkerke’s *R*^2^ = 0.11; *p* = 0.001; *n* = 246

**Table 5:** Logistic regression for depressive symptoms in physicians and nurses

	B	SE	*p*	OR	95% CI for OR	
Age < 40	0.394	0.345	0.254	1.483	0.753	2.919
Being female/diverse	**1.320**	**0.414**	**0.001**	**3.744**	**1.664**	**8.427**
Occupational setting			0.705			
Hospital setting	0.283	0.367	0.440	1.327	0.646	2.726
Other setting	0.420	0.759	0.580	1.522	0.344	6.742
Professional group nursing	0.225	0.360	0.531	1.253	0.618	2.538
Moral distress	**0.414**	**0.076**	** < 0.001**	**1.512**	**1.303**	**1.756**
Constant	–4.259	0.622	< 0.001	0.014		

Statistically significant values are printed in bold

*B* regression coefficient, *SE* standard error, *OR* odds ratio

Nagelkerke’s *R*^2^ = 0.32; *p* < 0.001; *n* = 243

**Table 6:** Logistic regression for burnout symptoms in physicians

	B	SE	*p*	OR	95% CI for OR	
Age < 40	**1.742**	**0.536**	**0.001**	**5.710**	**1.999**	**16.311**
Being female/diverse	–0.206	0.452	0.649	0.814	0.335	1.975
Hospital/other setting	0.017	0.446	0.970	1.017	0.424	2.436
Moral Distress	0.108	0.089	0.225	1.114	0.936	1.327
Constant	–0.962	0.514	0.061	0.382		

Statistically significant values are printed in bold

*B* regression coefficient, *SE* standard error, *OR* odds ratio

Nagelkerke’s *R*^2^ = 0.17; *p* = 0.008; *n* = 100

**Table 7:** Logistic regression for anxiety in physicians

	B	SE	*p*	OR	95% CI for OR	
Age < 40	1.104	0.597	0.064	3.017	0.936	9.721
Being female/diverse	–0.127	0.574	0.825	0.881	0.286	2.711
Hospital/other setting	0.076	0.586	0.897	1.079	0.342	3.401
Moral distress	**0.325**	**0.116**	**0.005**	**1.384**	**1.102**	**1.738**
Constant	–3.554	0.850	< 0.001	0.029		

Statistically significant values are printed in bold

*B* regression coefficient, *SE* standard error, *OR* odds ratio

Nagelkerke’s *R*^2^ = 0.16; *p* = 0.024; *n* = 117

**Table 8:** Logistic regression for depressive symptoms in nurses

	B	SE	*p*	OR	95% CI for OR	
Age < 40	0.243	0.490	0.620	1.275	0.488	3.335
Being female/diverse	**1.826**	**0.763**	**0.017**	**6.210**	**1.391**	**27.728**
Occupational setting			0.773			
Hospital setting	0.222	0.567	0.695	1.249	0.411	3.794
Other setting	0.702	0.993	0.480	2.018	0.288	14.138
Number of COVID-19 cases in employees	–0.123	0.220	0.576	0.884	0.575	1.361
Number of COVID-19 cases in patients	0.020	0.042	0.627	1.020	0.941	1.107
Increase in workload	**1.524**	**0.645**	**0.018**	**4.590**	**1.296**	**16.265**
Sufficient access to protective clothing	0.286	0.522	0.584	1.331	0.478	3.702
Feeling sufficiently protected from COVID-19	0.212	0.539	0.693	1.237	0.430	3.554
Staff shortages	0.569	0.528	0.281	1.767	0.627	4.977
Difficulties in building relationships with patients	0.430	0.470	0.360	1.537	0.612	3.861
Having been vaccinated at least once	0.036	0.681	0.958	1.037	0.273	3.936
Moral distress	**0.321**	**0.125**	**0.010**	**1.379**	**1.079**	**1.762**
Constant	–6.053	1.709	< 0.001	0.002		

Statistically significant values are printed in bold

*B* regression coefficient, *SE* standard error, *OR* odds ratio

Nagelkerke’s *R*^2^ = 0.34; *p* = 0.002; *n* = 112

**Table 9:** Logistic regression for burnout symptoms in nurses

	B	SE	*p*	OR	95% CI for OR	
Age < 40	0.682	0.528	0.197	1.978	0.702	5.569
Being female/diverse	–0.682	0.697	0.328	0.505	0.129	1.982
Occupational setting			0.413			
Hospital setting	–0.801	0.667	0.230	0.449	0.121	1.659
Other setting	0.166	1.048	0.874	1.181	0.151	9.210
Number of COVID-19 cases in employees	–0.038	0.214	0.859	0.963	0.634	1.464
Number of COVID-19 cases in patients	0.072	0.063	0.251	1.075	0.950	1.217
Increase in workload	0.950	0.657	0.148	2.585	0.714	9.365
Sufficient access to protective clothing	–0.648	0.542	0.232	0.523	0.181	1.515
Feeling sufficiently protected from COVID-19	–0.064	0.570	0.911	0.938	0.307	2.867
Staff shortages	0.588	0.595	0.323	1.800	0.561	5.779
Difficulties in building relationships with patients	0.367	0.528	0.487	1.443	0.513	4.059
Having been vaccinated at least once	0.193	0.721	0.789	1.213	0.295	4.983
Moral distress	**0.316**	**0.148**	**0.032**	**1.372**	**1.027**	**1.832**
Constant	–2.200	1.559	0.158	0.111		

Statistically significant values are printed in bold

*B* regression coefficient, *SE* standard error, *OR* odds ratio

Nagelkerke’s *R*^2^ = 0.31; *p* = 0.026; *n* = 93

**Table 10:** Logistic regression for moral distress in nurses

	B	SE	*p*	OR	95% CI for OR	
Age < 40	0.188	0.471	0.690	1.207	0.479	3.036
Being female/diverse	**1.317**	**0.613**	**0.031**	**3.734**	**1.0124**	**12.404**
Occupational setting			0.972			
Hospital setting	–0.099	0.545	0.857	0.906	0.311	2.640
Other setting	0.066	0.929	0.943	1.069	0.173	6.597
Number of COVID-19 cases in employees	0.347	0.309	0.261	1.414	0.772	2.590
Number of COVID-19 cases in patients	0.017	0.042	0.686	1.017	0.937	1.103
Increase in workload	1.021	0.540	0.059	2.776	0.964	7.995
Sufficient access to protective clothing	–0.080	0.514	0.876	0.923	0.337	2.526
Feeling sufficiently protected from COVID-19	**–1.314**	**0.550**	**0.017**	**0.269**	**0.091**	**0.790**
Staff shortages	0.152	0.488	0.755	1.164	0.447	3.030
Difficulties in building relationships with patients	0.618	0.455	0.175	1.854	0.760	4.522
Having been vaccinated at least once	0.736	0.649	0.257	2.087	0.585	7.449
Constant	–1.531	1.326	0.248	0.216		

Statistically significant values are printed in bold

*B* regression coefficient, *SE* standard error, *OR* odds ratio

Nagelkerke’s *R*^2^ = 0.29; *p* = 0.007; *n* = 114

## Discussion

Given country-specific differences regarding the course of the pandemic as well as the organization of health care, data from other countries cannot necessarily be extrapolated. This study, to the best of our knowledge, for the first time provides data on mental burden and moral distress of oncologists and oncology nurses working in Germany during the corona pandemic. Our data indicate a clinically relevant mental burden among a subgroup of participating physicians as well as nurses. Moderate-to-severe depressive symptoms were particularly pronounced in nurses and found to be associated with increase in workload in this profession as well as with being female/diverse and moral distress in both physicians and nurses. Additionally, being female was associated with higher moral distress in nurses, while feeling sufficiently protected from COVID-19 was associated with less moral distress.

### Prevalence of mental burden and moral distress

Compared to a nationwide cross-sectional study conducted among the German population (*n* = 15,704) during the first wave, which showed a prevalence of depressive symptoms (cutoff: PHQ-2 ≥ 3) and anxiety (cutoff: GAD-7 ≥ 10) of 14.3 and 16.8% respectively, physicians in our study showed a similar prevalence of depressive symptoms (19.2%) and anxiety (14.5%), while nurses displayed a higher prevalence of both depressive symptoms (41.4%) and anxiety (24.0%) (Bäuerle et al. 2020). Although comparability may be limited in some cases due to different survey instruments, our study in part showed similar results compared to data from international studies regarding prevalence of mental burden on healthcare workers (Helaß et al. 2022; Hilmi et al. 2020; Schneider et al. 2021; Thomaier et al. 2020; Varghese et al. 2021). However, oncologists in our study showed less anxiety than oncologists in the US (cutoff: PHQ-4 ≥ 3) during March and April 2020 (14.5% vs. 62.0%) and oncology residents working in France (cutoff: HADS ≥ 8) in May 2020 (14.5% vs. 32.0%) (Hilmi et al. 2020; Thomaier et al. 2020). The detected differences concerning anxiety are notable insofar as one might expect that burden may increase during the pandemic given the accumulated additional burden over time and little time for recovery. However, the subjective level of information regarding COVID-19 has been shown to be negatively associated with mental burden, including generalized anxiety symptoms (Bäuerle et al. 2020). Therefore, an explanation for the finding may be that physicians in our study at the time of the third wave felt sufficiently prepared for their tasks resulting in less anxiety.

In our study, nurses displayed higher levels of moral distress (5.09 ± 2.26; CI: 4.60–5.40) compared to nurses, who worked in hospital settings in the US before the COVID-19 pandemic (2.9 ± 2.5) as well as compared to nurses working in Norway during April/May 2020 (*M* = 3.1; CI 2.8–3.3.) (Miljeteig et al. 2021; Wocial and Weaver 2013). Compared to a German survey with 3293 health care professionals (*n* = 1149 nurses, *n* = 966 physicians), which was conducted between April and July 2020, nurses in our sample displayed higher scores of moral distress (5.09 ± 2.26 vs. 4.52 ± 2.66), while physicians showed a comparable result (3.83 ± 2.36 vs. 3.42 ± 2.61) (Schneider et al. 2021). Our results indicate that moral distress among oncology health care workers might have increased over the course of the pandemic and according to the duration of having to work under restricted conditions.

### Factors associated with mental burden and moral distress

Belonging to the nursing profession has been shown to be a risk factor for the development of mental disorders in the context of occupational stress during the pandemic (Kramer et al. 2021). In line with that, nurses in our study displayed higher mental burden than physicians with regard to all assessed mental health outcomes, especially moderate and moderately severe depressive symptoms. However, logistic regression did not show an association between profession and moderate to severe depressive symptoms for this sample.

Our results confirm the association between mental burden during the pandemic and being female/diverse, younger age, as well as higher workload shown in other studies (Banerjee et al. 2021; Bäuerle et al. 2020; Helaß et al. 2022; Petzold et al. 2020; Schmuck et al. 2022; Thomaier et al. 2020). Belonging to the nursing profession was significantly associated with moral distress and nurses displayed higher mean scores of moral distress than physicians, which is in line with previous studies (Mehlis et al. 2018; Pergert et al. 2019; Schneider et al. 2021). Furthermore, moral distress was associated with higher mental burden on physicians and nurses in this study. Being female was associated with higher moral distress in nurses, while nurses who felt sufficiently protected from COVID-19 experienced less moral distress. While there is scarce literature, the association between moral distress and mental burden as well as a positive association between moral distress and being female was also found in a review by Riedel et al. (Riedel et al. 2022). The literature is ambiguous regarding the influence of age and work experience on moral distress. However, younger age has been shown to favor moral distress in the context of the pandemic (Riedel et al. 2022). Our study showed no significant association between age and moral distress, which might be due to the relatively high median age and homogeneous age distribution in our sample. The results of the logistic regression analysis are to be considered robust in comparison with linear regression analysis. There is need to substantiate the current evidence on risk and protective factors for moral distress to be able to develop possible strategies to protect healthcare workers within and beyond the pandemic.

In this context, it is noteworthy, that the concept of moral distress is somehow elusive and its definition and operationalization are subject of debate. However, there is a certain consensus that “illegitimate constraints on individuals’ moral agency” constitute a hallmark of moral distress. Unfortunately, existing measurement tools cannot determine with certainty, whether the assessed subjective feeling of moral distress results from such an illegitimate constraint, and whether the distress experienced is correctly classified as moral (Kolbe and Melo-Martin 2022). Therefore, those tools do not allow a reliable normative judgment about the action(s) of a person. On the other hand, from a dialogical ethics point of view, subjective moral intuitions of stakeholders can be considered as relevant information and contribution to solving ethical issues (Abma et al. 2009). Furthermore, considering the negative impact moral distress can have on mental health and job satisfaction of healthcare workers, subjectively experienced moral distress might be of relevance regardless of its cause or normative evaluation (Kolbe and de Melo Martin 2022).

## Limitations

One important limitation of this study is the low response rate, which may have resulted in a sampling bias. Factors contributing to the low response rate could be due to the demanding work during the third wave or to the recruitment strategy via mailing lists. Furthermore, due to use of mailing lists provided by the German Cancer Society (nurses and oncologists), the findings cannot be extrapolated to all healthcare professions involved in cancer care. Since young age in particular seems to be associated with mental burden in health care workers and since the average age and work experience in this study were relatively high in both samples, mental burden may have been underestimated. Due to the limited data quality, the statistical analyses could only be interpreted in an exploratory manner. Results and possible conclusions described above are not generalizable, but may only be interpreted as indications and need to be verified in further studies. Nevertheless, under the circumstances, this recruitment strategy represented the best way to achieve the largest sample possible within a short period of time. Another limitation is the lack of pre-pandemic baseline data and follow-up data, which is due to the cross-sectional design. Therefore, no conclusions can be drawn about causal relationships between mental burden and other parameters examined in this study. It is also not possible to distinguish whether the high mental burden during the pandemic is associated with the pandemic in general or with specific challenges for health care workers. Furthermore, moral distress was assessed via a German version of the MDT, which has not been validated in itself, but is based on the validated German Version of the CDT (Mehlis et al. 2018; Mehnert et al. 2006).

## Conclusion

Our data point to a clinically relevant mental burden on oncologists as well as nurses in the context of the COVID-19 pandemic, which was associated with moral distress. Nurses, who felt sufficiently protected from COVID-19, experienced less moral distress. To improve pandemic resilience, there is a need to ensure safe working environment for healthcare workers including psychosocial support and to further substantiate the current evidence on risk and protective factors for moral distress to be able to develop and implement strategies to relieve this burden and thus protect healthcare workers.

## Supplementary Information

Below is the link to the electronic supplementary material.

Supplementary file1 (DOCX 27 KB)

## Data Availability

The datasets generated during this study are available from the corresponding author on reasonable request.
