# Supplementary material for: Mental burden and moral distress among oncologists and oncology nurses in Germany during the third wave of the COVID-19 pandemic: a cross-sectional survey
Source: J Cancer Res Clin Oncol. 2023 Jan 26;149(9):6211–23. doi: 10.1007/s00432-023-04580-x (PMC9878480; doi:10.1007/s00432-023-04580-x)
Supplement: Supplementary file 1 — Supplementary file1 (DOCX 27 KB) [file 432_2023_4580_MOESM1_ESM.docx]

**Journal of Cancer Research and Clinical Oncology**

**Mental burden and moral distress among oncologists and oncology nurses in Germany during the third wave of the COVID-19 pandemic. A cross-sectional survey**

**Sabine Sommerlatte^1*^, Celine Lugnier^2*^, Olaf Schoffer^3^, Patrick Jahn^4^, Anna-Lena Kraeft^2^, Eleni Kourti^2^, Patrick Michl^5^, Anke Reinacher-Schick^2^, Jochen Schmitt^3^, Thomas Birkner^3^, Jan Schildmann^1*^, Stephan Herpertz^6*^**

^1^ Faculty of Medicine, Interdisciplinary Centre for Health Sciences, Institute for History and Ethics of Medicine, Martin Luther University Halle-Wittenberg, Halle (Saale), Germany

^2^ Department of Hematology, Oncology and Palliative Care, St. Josef Hospital, Ruhr University Bochum, Bochum, Germany

^3^ Center for Evidence-Based Healthcare, University Hospital Carl Gustav Carus and Carl Gustav Carus Faculty of Medicine, Technische Universität Dresden, Dresden, Germany

^4^ Health Services Research Working Group, Department of Internal Medicine, University Hospital Halle (Saale), Halle (Saale), Germany

^5^ Department of Medicine, Internal Medicine IV, University Hospital Heidelberg, Heidelberg, Germany

^6^ Department of Psychosomatic Medicine and Psychotherapy, LWL-University Clinic, Ruhr University Bochum, Germany

*contributed equally

**Funding:** The project on which this report is based was funded by the German Federal Ministry of Education and Research (funding code 01KI20521A-C). The responsibility for the content of this publication lies with the authors.

**Correspondence to:**

Sabine Sommerlatte M. mel.

sabine.sommerlatte@medizin.uni-halle.de

ORCID: 0000-0001-6239-4349

**Supplementary Table 1** Linear regression for moral distress in physicians and nurses

|  | B | SE | Beta | p | 95.0% CI for B | |
| --- | --- | --- | --- | --- | --- | --- |
| Constant  Age < 40  Being female/diverse  Hospital/other setting  Other setting  **Professional group nursing** | 3.692 | 0.328 |  | <.001 | 3.046 | 4.339 |
|  | -0.366 | 0.330 | -0.072 | 0.269 | -1.015 | 0.284 |
|  | 0.354 | 0.337 | 0.072 | 0.295 | -0.310 | 1.018 |
|  | 0.135 | 0.328 | 0.027 | 0.681 | -0.512 | 0.782 |
|  | 0.158 | 0.753 | 0.014 | 0.834 | -1.326 | 1.642 |
|  | **1.148** | **0.330** | **0.241** | **<0.001** | **0.499** | **1.797** |

Statistically significant values are printed in bold
B regression coefficient, SE standard error, Beta standardized regression coefficient
Adjusted R^2^ = 0.06; p = 0.002; n = 246

**Supplementary Table 2** Linear regression for depressive symptoms in physicians and nurses

|  | B | SE | Beta | p | 95.0% CI for B | |
| --- | --- | --- | --- | --- | --- | --- |
| Constant  **Age < 40**  **Being female/diverse**  Hospital/other setting  Other setting  Professional group nursing  **Moral distress** | 1.092 | 0.786 |  | 0.166 | -0.456 | 2.640 |
|  | **1.296** | **0.639** | **0.116** | **0.044** | **0.037** | **2.554** |
|  | **2.078** | **0.656** | **0.193** | **0.002** | **0.786** | **3.370** |
|  | 0.007 | 0.638 | 0.001 | 0.991 | -1.249 | 1.264 |
|  | 0.117 | 1.445 | 0.005 | 0.936 | -2.730 | 2.963 |
|  | -0.015 | 0.652 | -0.001 | 0.982 | -1.299 | 1.269 |
|  | **0.998** | **0.124** | **0.458** | **<0.001** | **0.754** | **1.243** |

Statistically significant values are printed in bold
B regression coefficient, SE standard error, Beta standardized regression coefficient
Adjusted R^2^ = 0.28; p < 0.001; n = 243

**Supplementary Table 3** Linear regression for anxiety in physicians

|  | B | SE | Beta | p | 95.0% CI for B | |
| --- | --- | --- | --- | --- | --- | --- |
| Constant  **Age < 40**  Being female/diverse  Hospital/other setting  **Moral distress** | 0.524 | 1.331 |  | 0.695 | -2.115 | 3.162 |
|  | **2.472** | **0.843** | **0.244** | **0.004** | **0.802** | **4.142** |
|  | 0.704 | 0.729 | 0.080 | 0.336 | -0.741 | 2.149 |
|  | -0.473 | 0.733 | -0.053 | 0.520 | -1.925 | 0.980 |
|  | **0.855** | **0.150** | **0.461** | **<0.001** | **0.558** | **1.152** |

Statistically significant values are printed in bold
B regression coefficient, SE standard error, Beta standardized regression coefficient
Adjusted R^2^ = 0.25; p < 0.001; n = 100

**Supplementary Table 4** Linear regression for depressive symptoms in nurses

|  | B | SE | Beta | p | 95.0% CI for B | |
| --- | --- | --- | --- | --- | --- | --- |
| Constant  Age < 40  **Being female/diverse**  Hospital setting  Other setting  Number of COVID-19 cases in employees  Number of COVID-19 cases in patients  **Increase in workload**  Sufficient access to protective clothing  **Feeling sufficiently protected from COVID-19**  Staff shortages  Difficulties in building relationships with patients  Having been vaccinated at least once  **Moral distress** | -3.472 | 3.471 |  | 0.320 | -10.361 | 3.416 |
|  | -0.126 | 0.861 | -0.012 | 0.884 | -1.835 | 1.583 |
|  | **3.038** | **1.134** | **0.227** | **0.009** | **0.787** | **5.289** |
|  | -0.407 | 0.987 | -0.039 | 0.681 | -2.366 | 1.552 |
|  | -0.251 | 1.733 | -0.013 | 0.885 | -3.690 | 3.188 |
|  | -0.012 | 0.379 | -0.003 | 0.974 | -0.764 | 0.739 |
|  | 0.023 | 0.064 | 0.030 | 0.718 | -0.105 | 0.151 |
|  | **2.910** | **1.034** | **0.262** | **0.006** | **0.859** | **4.961** |
|  | 0.269 | 0.926 | 0.026 | 0.772 | -1.568 | 2.106 |
|  | -0.644 | 0.970 | -0.063 | 0.508 | -2.568 | 1.280 |
|  | 10.489 | 0.915 | 0.144 | 0.107 | -0.326 | 3.305 |
|  | 0.739 | 0.842 | 0.075 | 0.383 | -0.933 | 2.410 |
|  | 0.038 | 1.198 | 0.003 | 0.975 | -2.340 | 2.415 |
|  | **0.693** | **0.209** | **0.306** | **0.001** | **0.279** | **1.108** |

Statistically significant values are printed in bold
B regression coefficient, SE standard error, Beta standardized regression coefficient
Adjusted R^2^ = 0.29; p < 0.001; n = 112

**Supplementary Table 5** Linear regression for moral distress in nurses

|  | B | SE | Beta | p | 95.0% CI for B | |
| --- | --- | --- | --- | --- | --- | --- |
| Constant  Age < 40  Being female/diverse  Hospital setting  Other setting  Number of COVID-19 cases in employees  Number of COVID-19 cases in patients  **Increase in workload**  Sufficient access to protective clothing  **Feeling sufficiently protected from COVID-19**  Staff shortages  **Difficulties in building relationships with patients**  Having been vaccinated at least once | 1.490 | 1.571 |  | 0.345 | -1.626 | 4.607 |
|  | 0.448 | 0.401 | 0.100 | 0.266 | -0.347 | 1.243 |
|  | 1.023 | 0.515 | 0.175 | 0.050 | 0.001 | 2.046 |
|  | 0.158 | 0.457 | 0.034 | 0.731 | -0.749 | 1.065 |
|  | 0.622 | 0.822 | 0.073 | 0.451 | -1.008 | 2.253 |
|  | 0.186 | 0.180 | 0.092 | 0.302 | -0.170 | 0.543 |
|  | -0.011 | 0.031 | -0.032 | 0.714 | -0.072 | 0.050 |
|  | **0.972** | **0.482** | **0.197** | **0.046** | **0.016** | **1.929** |
|  | -0.184 | 0.432 | -0.040 | 0.671 | -1.042 | 0.674 |
|  | **-10.078** | **0.446** | **-0.238** | **0.017** | **-1.962** | **-0.194** |
|  | 0.188 | 0.432 | 0.041 | 0.664 | -0.669 | 1.045 |
|  | **0.895** | **0.389** | **0.206** | **0.023** | **0.125** | **1.666** |
|  | 0.490 | 0.569 | 0.076 | 0.392 | -0.640 | 1.620 |

Statistically significant values are printed in bold
B regression coefficient, SE standard error, Beta standardized regression coefficient
Adjusted R^2^ = 0.17; p = 0.001; n = 114
